# Gamma-Glutamyl Transferase: A Friend against Cholestatic Itch? A Retrospective Observational Data Analysis in Patients with Extrahepatic Cholestasis

**DOI:** 10.1155/2023/2903171

**Published:** 2023-02-08

**Authors:** Floris W. Haijer, Cornelis B. Van Vliet, Marjolein G. J. Brusse-Keizer, Job A. M. Van der Palen, Marjo J. Kerbert-Dreteler, Jeroen J. Kolkman

**Affiliations:** ^1^Department of Gastroenterology and Hepatology, Medical Spectre Twente, Netherlands; ^2^Department of Statistics, Medical Spectre Twente, Netherlands

## Abstract

**Methods:**

We included 235 patients with chronic extrahepatic cholestasis due to pancreatic cancer, cholangiocarcinoma, or papillary carcinoma.

**Results:**

GGT was significantly higher in patients without pruritus (median 967, IQR 587–1571) compared to patients with pruritus (median 561 IQR 266–1084 IU/l) (*p* < 0.01). In contrast, median alkaline phosphatase (AP) was 491 U/L (IQR; 353–684) in patients with pruritus and was not significantly different from 518 U/L (IQR; 353–726) in patients without pruritus (*p* = 0.524). Direct bilirubin was significantly higher in patients with pruritus compared to patients without pruritus (168 *μ*mol/L (IQR; 95–256) vs. 120 *μ*mol/L (IQR; 56.75–185.5)) (*p* < 0.01). After correcting for the extent of cholestasis *via* direct bilirubin, the negative association between GGT and pruritus remained significant and became stronger (*p* < 0.001).

**Conclusion:**

Serum GGT activity is inversely associated with the presence of cholestatic itch in patients with chronic extrahepatic cholestasis.

## 1. Introduction

Chronic cholestasis can lead to pruritus, which occurs in approximately 45% of patients with cholestasis due to malignant obstructions [1]. However, it remains elusive why some cholestatic patients develop pruritus while other ones do not. During the last decades, important progress has been made in understanding the complex pathogenesis of cholestatic itch. Clinical observations have led to the conclusions that potential pruritogens accumulate in the systemic circulation, are (biotrans-)formed in the liver and/or gut, are secreted into bile, and affect the endogenous opioidergic and serotoninergic system [2]. A decade ago, lysophosphatidic acid (LPA) was identified as a possible mediator of cholestatic pruritus [3]. LPA is mainly formed by the lysophospholipase D activity of autotaxin (ATX), and high plasma ATX activity is associated with a higher prevalence of cholestatic itch [3]. However, plasma ATX activity is also elevated in patients without itch that suffer from hepatic diseases such as hepatitis B, hepatitis C, and nonalcoholic fatty liver disease [4]. Therefore, ATX might be only a biomarker instead of an enzyme that is causally linked to cholestatic itch.

Serum bile acid and bilirubin concentrations are positively associated with the presence of itch during cholestasis [3, 5–8]. Recently, the Mas-related G protein-coupled receptor X4 (MRGPRX4) has been identified as a bile acid and bilirubin receptor. Activation of MRGPRX4 induces itch in healthy volunteers and therefore MRGPRX4 activation by bile acids or bilirubin can possibly lead to cholestatic itch. However, patients with congenital diseases that lead to conjugated or unconjugated hyperbilirubinemia generally do not have itch [9]. In addition, there is no direct correlation between the serum bile acid concentration and the severity of cholestatic itch [3, 5, 10]. Furthermore, in patients with intrahepatic cholestasis of pregnancy (ICP), itch is highly prevalent. In these patients, pruritus begins approximately 3 weeks before serum bile acids are elevated [6, 7]. Therefore, the presence or absence of pruritus in clinical patients cannot be predicted and explained based on only plasma bilirubin and/or bile acid concentrations.

The cornerstone in the treatment of cholestatic itch is restoring bile flow. In extrahepatic cholestasis, this is frequently achieved by bile drainage procedures such as endoscopic retrograde cholangiopancreatography (ERCP) or percutaneous transhepatic cholangiography drainage (PTCD) [8, 11]. When adequate biliary drainage is not possible, patients are dependent on anti-pruritus medication. However, the effectiveness of current anti-pruritus medication is often limited [12–14], and there is a clear demand for novel drugs.

Identification of differences between cholestatic patients that have pruritus and similar cholestatic patients that do not have pruritus could unravel a protein or pathway that can be used for development of novel drugs. Clinical observations in a small number of patients suggested that high serum GGT activity, relative to bilirubin (as a marker for the extent of cholestasis), may be associated with a lower prevalence of cholestatic itch.

GGT is a protein that catalyzes transpeptidation of the glutamyl moiety of glutathione, an important antioxidant [15]. GGT is a well-known biomarker for cholestasis. However, GGT has not yet been linked to a specific function in cholestasis such as prevention of cholestatic itch.

The primary research question of the present study is: is high serum GGT activity, relative to the serum bilirubin concentration, associated with a lower prevalence of cholestatic itch?

## 2. Methods

### 2.1. Data Source

The data used in this study were obtained from medical records of patients that were admitted to the Medical Spectre Twente (MST) hospital of Enschede, The Netherlands, between 1-1-2015 and 31-12-2018.

In brief, patients were identified *via* diagnosis treatment codes (DTC) “pancreatic neoplasia” or “cholangiocarcinoma” that are used in the Netherlands for reimbursement purposes. Records were screened for in- and exclusion criteria and the presence of itch.

### 2.2. Study Design

We conducted a retrospective observational data analysis.

### 2.3. Inclusion Criteria

Adult patients (18 years of age or older) who were diagnosed with pancreatic cancer, cholangiocarcinoma, or papillary carcinoma were included. The presence of cholestasis was defined as elevated AP (>125 U/L), elevated direct bilirubin (>17 *μ*M/L), and GGT (>50 U/L). Patient history and laboratory measurements were conducted before interventions, such as endoscopic retrograde cholangiopancreatography (ERCP), percutaneous transhepatic cholangiodrainage (PTCD), pylorus preserving pancreaticoduodenectomy (PPPD), or chemotherapy.

### 2.4. Exclusion Criteria

Exclusion criteria were [1] active primary dermatological abnormalities associated with pruritus: xerosis, atrophic dermatitis, psoriasis, cutaneous infections, and cutaneous T-cell lymphoma; [2] recent allergic reactions; [3] active systemic diseases that are associated with pruritus; [4] chronic renal failure (defined as an eGFR <30 ml/min calculated *via* the MDRD (modification of diet in renal disease) formula), hematological, or lymphoproliferative diseases, [9] neurological diseases: notalgia paresthetica, brachioradial itching, and multiple sclerosis. Patients that used anticonvulsants such as gabapentin were not excluded; [5] active psychiatric disease associated with pruritus: depression, anxiety disorders, delusional parasitosis, and psychogenic excoriations; and [10] use of anti-pruritic drugs (cholestyramine, rifampicin, and naltrexone).

### 2.5. Research Questions

Primary research question: is a high GGT, relative to direct bilirubin, associated with a lower prevalence of cholestatic itch in patients with chronic extrahepatic cholestasis?

Secondary research questions: are serum GGT activity, alkaline phosphatase activity, or direct bilirubin different between patients with and without cholestatic pruritus?

### 2.6. Statistical Analysis

The association between GGT and pruritus was corrected for direct bilirubin with the use of logistic regression. Dichotomous data are presented as numbers and percentages. Continuous data are expressed as means and standard deviations (SD) or median with interquartile range (IQR), as appropriate. Between group differences in continuous variables were analyzed using independent *T*-tests or Mann–Whitney *U* test, as appropriate. Differences in categorical variables between groups were analyzed with Chi-squared test. All statistical test were two-sided with a significance level of 0.05. We used SPSS version 25.

## 3. Results

**Figure 1:**
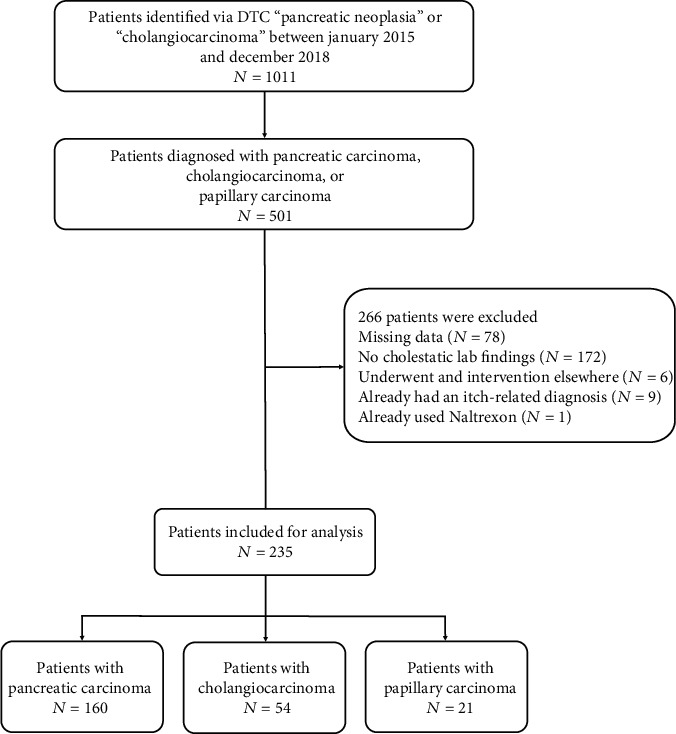
A schematic overview of included patients.

A total of 1011 patient records were screened based on diagnosis treatment codes, leading to 501 patients with pancreatic cancer, cholangiocarcinoma, or papillary cancer that were screened for eligibility (Figure 1).

266 patients did not meet inclusion criteria or met exclusion criteria. 78 patients were excluded because of missing data, mostly because they were only referred to our hospital for a drainage procedure. 172 patients did not have cholestatic laboratory findings. Six patients already underwent an intervention before presenting to our hospital, and 9 patients already had an itch related diagnosis. One patient was excluded because of the use of Naltrexone is a drug that is used against cholestatic itch, because of alcohol dependence [16]. 235 patients were included for analysis. 160 patients had pancreatic cancer, 54 patients had cholangiocarcinoma, and 21 patients had papillary carcinoma.

Patient characteristics are listed in Table 1. Pruritus was present in 99 of 235 (42.1%) patients. Transaminases and INR were significantly higher in patients without pruritus.

**Figure 2:**
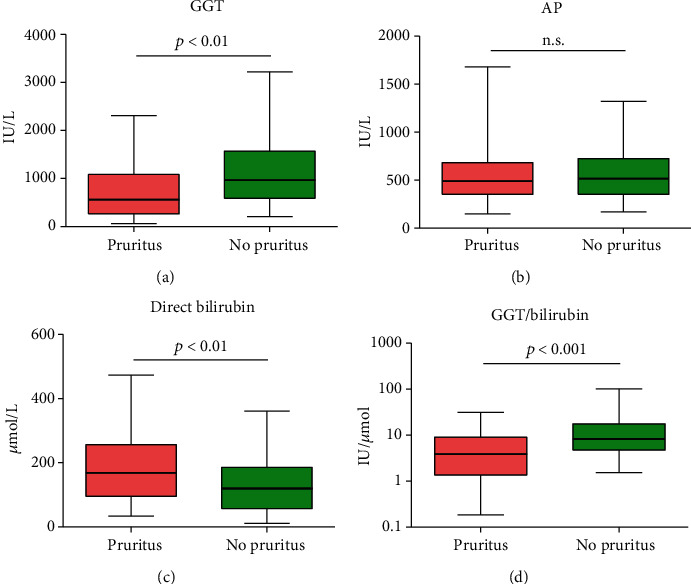
(a) Serum GGT activity in patients with and without pruritus. (b) Serum AP activity in patients with and without pruritus. (c) Serum direct bilirubin concentration in patients with and without pruritus. (d) Serum GGT divided by direct bilirubin. Whiskers present 2.5–97.5 percentiles.

Median serum GGT activity was 967 IU/L (IQR; 587–1571) in patients without pruritus, which was significantly higher compared to 561 IU/L (IQR; 266–1084) in patients with pruritus (*p* < 0.01) (Figure 2(a)).

Median serum AP was 518 U/L (IQR; 353–726) in patients without pruritus and was 491 U/L (IQR; 353–684) in patients with pruritus (*p* = 0.524) (Figure 2(b)). Median direct bilirubin was 120 *μ*mol/L (IQR; 56.75–185.5) in patients without pruritus and was 168 *μ*mol/L (IQR; 95–256) in patients with pruritus (*p* < 0.01) (Figure 2(c)). After correction for direct bilirubin, with the use of logistic regression, serum GGT activity was significantly higher in patients without pruritus compared to patients with pruritus (*p* < 0.001) (OR; 1.001).  Figure 2(d) presents a histogram of the GGT/bilirubin ratio.

## 4. Discussion

In this study, we confirm our hypothesis that high serum GGT activity, relative to direct bilirubin, is associated with a reduced prevalence of itch in patients with chronic extrahepatic cholestasis. Also, the absolute serum GGT activity was negatively associated with the presence of cholestatic itch. There was no difference in AP activity between itch and nonitch patients. Direct bilirubin was significantly higher in patients with pruritus.

GGT is a routinely requested serum parameter, and GGT data are available in many other studies that focused on cholestatic itch [3, 5, 8, 17–22]. However, in these studies, an association between cholestatic itch and GGT has not been reported. This can be due to several study-specific issues. First, many authors only included patients with itch, thus, making a comparison with findings in nonitching patients impossible [19–22]. In our study, we selected patients based on the presence of cholestasis which led to inclusion of patients with and without itch.

Second, the prevalence of cholestatic itch is dependent on the cause of cholestasis [1]. Therefore, the cause of itch can be a confounding factor when characteristics of patients with and without cholestatic itch are studied. For example, in a paper by Kremer et al., no clear difference in serum GGT activity between patients with and without itch was found [3]. However, in this paper, the diagnosis ranged from intrahepatic cholestasis of pregnancy (ICP) to hepatitis C. It may very well be that patients with itch generally had other causes of cholestasis compared to patients without itch. In addition, GGT levels were far lower than in our study (a median GGT activity of 151 IU/L and 186 IU/L in itching and nonitching subjects, respectively). The same group published another study describing the role of autotaxin and pruritus in children [17]. In contrast to our findings, GGT was higher in patients with pruritus in this study. However, the group of patients without pruritus only contained children with bile acid synthesis defects, while the group of patients with pruritus contained patients with Alagille syndrome and other cholestatic syndromes. Therefore, the difference in prevalence of cholestatic itch could probably be attributed to the difference in causes of cholestasis. In our study, we restricted the inclusion to patients with extrahepatic cholestasis due to malignant obstruction and therefore, and all patients had a similar cause of cholestasis.

Third, the extent of cholestasis is positively associated with the prevalence of cholestatic itch [3, 5, 8, 17, 18]. Therefore, the extent of cholestasis can be an important confounder. In our study, we corrected for the extent of cholestasis via direct bilirubin.

Taken together, this study focused on the association between GGT and cholestatic itch as a primary endpoint.

Our finding is in line with observations from relatively rare hepatic diseases that result in cholestasis with a low plasma GGT activity such as progressive familial intrahepatic cholestasis type 2 (PFIC2) and intrahepatic cholestasis of pregnancy. PFIC2 patients have a genetic defect in the ABCB11 gene which results in malfunction of the bile salt export pump (BSEP). BSEP is a transport protein that exports bile acids across the canalicular membrane of hepatocytes. These patients typically have severe cholestasis, severe itch, and low plasma GGT activity [23, 24].

Similar observations can be made in pregnant women with intrahepatic cholestasis of pregnancy (ICP). In ICP, itch without a rash generally is the primary presenting complaint [25]. Patients with ICP have cholestasis with a relatively low GGT (mean 22 UI/L; SD 17 IU/L) [26] [25, 27].

The strength of our study is that we studied the association between GGT and cholestatic itch in a selected patient cohort, and that we corrected for the extent of cholestasis as this was an important confounding factor. Weaknesses include the retrospective nature and the lack of quantification of itch severity.

An important question that follows from our data is whether GGT could be causally linked to cholestatic itch. Here, we speculate about two possible mechanisms. The main substrate for GGT is glutathione, the major antioxidant in human cells. A low glutathione concentration (4-8 *μ*M) is also present in blood plasma of healthy volunteers [27]. GGT is able to catalyze the transfer of the glutamyl moiety of glutathione to an acceptor to promote water solubility to enhance renal excretion [28, 29]. Possibly, GGT promotes the transfer of the glutamyl moiety of glutathione to pruritogens in order to inhibit their pruritogenic potential or promote water solubility to enhance renal excretion.

Another possibility is that GGT inhibits itching via synthesis of glutamine. GGT is able to cleave the glutamyl moiety of glutathione, which, in contact with water, can transform to glutamate. In liver and skeletal muscle tissue, glutamate can be coupled to ammonia to form glutamine [30]. Topical glutamine administration was shown to inhibit scratch behavior in an animal model of allergic contact dermatitis via the G-protein coupled receptor kinase (GRK-2) [31]. Therefore, in the presence of ammonia and glutathione, GGT possibly attenuates pruritus. Whether GRK-2 or GGT is involved in MRGPRX4 signaling is still unknown.

## 5. In Conclusion

The present study shows a strong negative association between serum GGT activity and the presence of itch in cholestatic patients.

The main question that rises from our results is whether this is a causal relationship or not. Therefore, our future perspective is to test GGT administration in an animal model of cholestatic itch.

## Figures and Tables

**Table 1:** Demographic and laboratory characteristics.

Characteristic	Patients without pruritus (*N* = 136)	Patients with pruritus (*N* = 99)	*p* value or mean difference (95% CI)
Gender—females (%)	65 (48)	41 (41)	0.33
Age in years (mean ± SD)	70.0 ± 10.7	69.0 ± 10.6	0.45 (-3.85–1.72)
Diagnosis—number (%)			
Pancreatic carcinoma	100 (73)	60 (61)	0.06
Cholangiocarcinoma	28 (21)	26 (26)	
Papillary carcinoma	8 (6)	13 (13)	
Hb—mmol/L (mean ± SD)	7.9 ± 1.1	8.1 ± 0.9	0.22 (-0.10–0.44)
Leucocytes—10^9/L (mean ± SD)	8.8 ± 3.9	8.3 ± 2.6	0.31 (-1.48–0.47)
Thrombocytes—10^9/L (mean ± SD)	295 ± 92	316 ± 91	0.13 (-6.31–48.0)
AST—U/L median (IQR)	162 (100–247)	114 (67–199)	<0.01
ALT—U/L median (IQR)	260 (129–407)	197 (78–320)	<0.01
LD—U/L median (IQR)	237 (201–284)	239 (203–274)	0.93
INR median (IQR)	1.1 (1.00–1.30)	1.0 (0.975–1.10)	<0.01
Albumin—g/L (mean ± SD)	31.3 ± 6.1	31.3 ± 4.8	0.99 (-2.21–2.17)
MELD-score (mean ± SD)	17.7 ± 5.2	18.5 ± 4.2	0.47 (-1.37–2.93)
Creatinine—*μ*mol/L (mean ± SD)	74 ± 49.2	73 ± 25.4	0.98 (-11.6–11.2)
eGFR (MDRD) —ml/min (mean ± SD)	97 ± 38.4	92 ± 30.0	0.32 (-14.7–4.83)

Abbreviations: Hb: hemoglobin; AST: aspartate aminotransferase; ALT: alanine aminotransferase; LD: lactate dehydrogenase; INR: international normalized ratio; MELD: model for end-stage liver disease; eGFR: estimated glomerular filtration rate; MDRD: modification of diet in renal disease.

## Data Availability

All methods that were used are described in the methods section. Data of this manuscript are available upon reasonable request to the corresponding author.

## Abbreviations

GGT: Gamma-glutamyl transferase
U/L: Units per liter
LPA: Lysophosphatidic acid
ATX: Autotaxin
ERCP: Endoscopic retrograde cholangiopancreatography
PTCD: Percutaneous transhepatic cholangiography drainage
Hb: Hemoglobin
AST: Aspartate aminotransferase
ALT: Alanine aminotransferase
LD: Lactate dehydrogenase
INR: International normalized ratio
MRGPRX4: Mas-related G protein-coupled receptor X4
MELD: Model for end-stage liver disease
eGFR: Estimated glomerular filtration rate
MDRD: Modification of diet in renal disease
GRK-2: G-protein coupled receptor type 1.
ICP: Intrahepatic cholestasis of pregnancy.
PFIC2: Progressive familial intrahepatic cholestasis type 2
IQR: Interquartile range
SD: Standard deviation.
